# Surveillance of tobacco use among young adolescents: trends and predictors across three years in Sousse, Tunisia

**DOI:** 10.1186/s12889-022-14416-x

**Published:** 2022-11-04

**Authors:** Nawel Zammit, Jihene Maatoug, Rim Ghammam, Sihem Ben Fredj, Wafa Dhouib, Imen Ayouni, Amani Maatouk, Waad Ben Belgacem, Mohamed Ouertani, Hassen Ghannem

**Affiliations:** 1 Department of Epidemiology (LR19SP03), Farhat Hached University Hospital, 4000 Sousse, Tunisia; 2 Faculty of Medicine of Sousse, University of Sousse, 4000 Sousse, Tunisia; 3 Department of Epidemiology, Farhat Hached University Hospital, 4000 Sousse, Tunisia

**Keywords:** Epidemiological monitoring, Tobacco use, Adolescent

## Abstract

**Background:**

In developed countries, there was an overall decrease in tobacco use over the last decades. In Tunisia, a national strategy to reduce tobacco use was set up since 2008. However, this strategy was rarely evaluated. The objective of the current study was to examine the trends in tobacco use among the middle schoolchildren of the governorate of Sousse (Tunisia) between 2014 and 2016 and to determine predictors of its experimentation.

**Methods:**

Three cross-sectional studies were conducted in 2014, 2015 and 2016 school years among middle schoolchildren randomly selected from the governorate of Sousse-Tunisia. The required sample size for each study was 760 participants. Each year, the same procedure was used to recruit pupils from the same middle schools. The same pre-established and pre-tested questionnaire was self-administered anonymously to participants in their classrooms.

**Results:**

Lifetime tobacco use rose from 11% in 2014 to 17.3% in 2016 (*p* = 0.001). Across the 3 years of survey, predictors of lifetime tobacco use were: The male sex (OR, 95% CI: 4.4 [3.2-6.1]), age above 13 (OR, 95% CI: 2.3 [1.7-3.1]), lifetime illicit substances use (OR, 95% CI: 3.9 [1.1- 13.8), lifetime inhalant products use (OR, 95% CI: 2.2 [1.2-4.3]), tobacco use among the father (OR, 95% CI: 2.2 [1.2-4.3]), tobacco use among siblings (OR, 95% CI: 1.7 [1.2-2.4]) and current anxiety symptoms (OR, 95% CI: 1.8 [1.4-2.4]).

**Conclusion:**

Lifetime tobacco use is in expansion among the young adolescents of Sousse. The current national tobacco prevention program should be strengthened and expanded to cover other substances use issues with emphasis on secondhand smoking and mental health problems.

## Introduction

Globally, one in every 5 boys and one in every 10 girls aged between 13 and 15 years use tobacco and these rates are rising steadily [1, 2]. In fact, onset of tobacco use usually coincides with early adolescence. This early exposition may result in a longer time of exposure to the constituents of tobacco products [2–4]. Besides, the higher vulnerability to nicotine addiction among adolescents may increase the risk of regular tobacco consumption and a heavier use of tobacco during adulthood [2–4]. Consequently, significant health problems may occur during adulthood such as cardiovascular diseases, chronic obstructive pulmonary disease and cancers [5–7]. Indeed, without preventive measures, the half of teenage smokers will die in adulthood from tobacco-related diseases [8].

Several other risk behaviors usually emerge during early adolescence and tend to cluster with tobacco use such as substances use, mental health problems, risky sexual behavior, injury risk behavior like motor vehicle accidents and acts of violence in addition to sedentary behavior and unhealthy eating habits [2, 3, 9]. The gateway effect of tobacco in addition to common socio-cultural and psycho-pathological risk factors may be behind these risk behaviors [10, 11].

In developing countries, which have not yet completed their epidemiological transition and where 90% of young people live, there is a lack of research studies about tobacco use among adolescents [12–14]. In Tunisia, a national strategy to reduce tobacco use was adopted since 2008. However, several components of this strategy were not implemented especially legislative ones. Furthermore this strategy was rarely evaluated [15]. Recently, in the governorate of Sousse, an urban area located at the center of Tunisia, a prevention program was implemented between 2010 and 2013 in order to promote healthy lifestyle in middle schools, workplaces and community settings [16]. The short-term evaluation did not show significant decrease of tobacco use among middle school children. Indeed, the political instability did not allow the implementation of the required multisectoral actions [17]. Epidemiological surveillance of tobacco use among Tunisian young adolescents will contribute to a better understanding of this health issue and guide accordingly its future preventive actions [10, 12]. Indeed, surveillance of Tobacco use is a measure that was recommended to fight against the global tobacco epidemic [18].

The objective of the current study was to examine the trends in tobacco use among the middle school children of the governorate of Sousse, Tunisia between 2014 and 2016 and to determine predictors of its experimentation.

## Methods

### Study design

Three cross-sectional studies were conducted in the governorate of Sousse, Tunisia successively during the months of April and May in 2014, 2015 and 2016 to monitor tobacco use among middle school pupils.

### Participants

In 2014, the population size was at 674818 in the governorate of Sousse [19]. People aged between 10 and 14 years represented 6.9% [19]. According to the regional direction of education, there is 40 public middle schools (with 31,844 pupils enrolled) and 15 private schools in Sousse. Usually, registration fees in the private schools are expansive, accordingly, most pupils enrolled there, are with high socioeconomic level. To ovoid this selection bias and for feasibility considerations, the source population was extracted from public middle schools. The number of pupils in these schools varies between 306 and 1370.

Sample size calculation was based on the following formula [20]: n = (p-q) Z_α_^[2]^ /i^2^ with an expected prevalence (p) of tobacco use of 12.9% [21], q = 1-p, a precision (i) of 2.5%, a confidence level of 95% and a non-response rate of 10%. In total a sample size of 760 participants was required. In order to reduce the dispersion of the sample, four middle schools were randomly selected from the 40 of the governorate of Sousse. They included 525, 860, 1370 and 1142 pupils. According to the number of pupils’ classes in each of the four middle schools and their repartition by study level, a cluster sampling with probability proportional to size was performed to select pupils’classes in 2014. In the next 2 years, the same procedure was used to recruit pupils from the same middle schools. All pupils in these classes that were present during data collection, provided written parental consent and accepted to participate were included in the study.

### Data collection

The same questionnaire that was previously designed in Arabic language to evaluate the effectiveness of the intervention program conducted in Sousse between 2010 and 2013 [16], was used to collect data among the participants of 2014, 2015 and 2016. It was prepared based on a literature review and validated by a group of multidisciplinary experts in Public Health and Family Medicine to ensure that the questions are clear, relevant and in line with the objective of the study. It contains sections exploring sociodemographic characteristics, stress, mood and sleep problems, smoking status, tobacco use among family members, substances use among participants and in the entourage. This questionnaire was pre-tested on a convenience sample of 30 adolescents to assess the cultural acceptability and the understandability of the items. Unclear items and those that were difficult to understand by 2 or more adolescents were reformulated considering their comments and the opinion of experts. It requires 30 to 40 minutes to be filled in. It was self-administered anonymously to the participants in their classrooms and in the presence of pre-trained medical doctors.

Concerning self-reported smoking status, in 2014, it was assessed among a sample of 147 schoolchildren randomly selected from another middle school using a breath Carbon Monoxide monitor (piCO+Smokerlyzer®) using sensitivity and specificity as accuracy measures. These latter were at 100 and 93.7% respectively with a cut-off of 4 ppm and at 62.5 and 93.5% with a cut-off of 3 ppm [22].

### Study variables

-Lifetime tobacco use was assessed using the following question “Have you ever smoked a cigarette or tried to use a tobacco product even once in your life? Lifetime tobacco use was defined as using a tobacco product at least once a life.

-Current cigarette smoking was assessed using the following question “During the last month, how many days did you smoke cigarettes?”. A current smoker was defined as a person who smoked at least one cigarette in the previous month.

–Lifetime use of illicit substances was assessed using the following question “Have you ever used a psychoactive substance even once in your life?” Possible responses were “No”, “Cannabis”, “Trihexyphenidyl/Artane”, “Cocaine”, “Heroine”, “Fentanyl”, “Ecstasy”, “Subutex”, “Madkok”, “LSD”, “Ketamine” and “other substances”. Lifetime use of illicit substance was defined as using at least one of these substances at least once a life.

-Lifetime use of inhalant products was assessed using the following question “Have you ever used an inhalant product (glue, paint, paint thinner, …) even once in your life? Lifetime use of inhalant products was defined as using it at least once a life.

-Current mental health problems were evaluated using the questions “During the last 6 months, how many times you felt that you were sad or anxious or stressed or you had insomnia?”. Response categories to these questions were: “rarely or never”, “every month”, “every week”, “more than once a week” and “every day”. The responses were then dichotomized as following: “every week”, “more than once a week” and “every day” were re-coded to “yes” and the other responses were re-coded to “no” to define either the participant had or not the symptom at least once a week.

### Data analysis

Data capture and analysis were performed using SPSS for Windows 10.0 (SPSS Inc., Chicago, IL, USA). Descriptive statistics were reported as frequencies for categorical variables and as means and standard deviations for quantitative ones. Differences between groups were examined using the Student’s t-test to compare means and the Chi-squared (χ^2^) test to compare proportions. When the application criteria for this latter were not met, the Fisher test was used.

Evolution of substances use frequencies across the 3 years of survey was assessed using univariate logistic regression models. The dependent variables had two modalities: “yes” and “no”. The variable “survey year” was introduced as an independent variable in each model. It had three modalities: “2014”, “2015” and “2016”. The modality “2014” was the reference. Accordingly, comparisons were performed between the 2015 and 2014 survey years in one hand and between 2016 and 2014 on the other hand.

To determine factors associated with the dependent variable “lifetime tobacco use” across the 2 years of survey, binary logistic regressions were performed. In each model, only one independent variable was introduced with the variable “survey year”. The 2014 survey year was kept as a reference. The magnitude of the association between each independent variable and the variable “lifetime tobacco use” across 2014, 2015 and 2016 was estimated by calculating the OR according to the method of Mantel Hanzel.

To determine the most influencing factors on lifetime tobacco use, all factors that were revealed to be associated with lifetime tobacco use across the 3 years of survey with a significance level less than 25% were included in a multivariable model with the variable “survey year”. The 2014 survey year was kept as a reference. The interaction terms with a significance level less than 25% were also included in the multivariable model. Then, a stepwise backward approach was used to identify predictors of lifetime tobacco use.

Observations with missing data about variables that were used in the different regression models were deleted. The Hosmer-Lemeshow test served to check for the adequacy of the final model.

Results of the regression models were expressed as odds ratios (ORs) with confidence interval (CI) of 95%. All statistical tests were two-tailed, and *p*-values < 0.05 were considered statistically significant.

### Ethical considerations

The current study was carried out in accordance with the ethical principles of the Declaration of Helsinki. It was approved by the Ethical Committee of Farhat Hached University Hospital (Institutional review board code: 00008937). Authorizations were obtained from the Regional Education Direction and the directors of the four schools. In addition, an information form was previously distributed to the parents of the participants in order to explain the purpose and the conduct of the study. Parents were free to refuse their child’s participation. Before the administration of the questionnaire in the classrooms, the investigators requested permission from teachers and pupils and explained the anonymity of the participation.

### Results

No significant difference was observed concerning the distributions of participants according to the sex or the age across the three surveys except for the proportion of pupils above 13 years in 2016 which was significantly higher than that in 2014 (Table 1).

**Table 1:** Characteristics of the participants of 2014, 2015, and 2016

Characteristics	2014 (***n*** = 751)		2015 (*n* = 751)		2016 (***n*** = 715)		Comparison between 2014 and 2015	Comparison between 2014 and 2016
	n (%)	[95% CI]	n (%)	[95% CI]	n (%)	[95% CI]	***p*** value	*p* value
**Sociodemographic characteristics**								
**Males**	365 (48.7)	45.1, 52.3	346 (46.9)	43.3,50.5	323 (46.7)	43.0,50.4	0.419	0.415
**Age > 13 years**	325 (43.4)	39.9,46.9	354 (47.5)	43.9,51.1	354 (49.9)	46.2,53.6	0.120	0.013^b^
**Lifetime tobacco use**	82 (11.0)	8.8,13.2	92 (12.5)	10.1,14.9	123 (17.3)	14.5,20.1	0.358	0.001
**Male**	68 (18.7)	15.9,21.5	70 (20.6)	17.7,23.5	78 (24.5)	21.3,27.6	0.536	0.070
**Female**	14 (3.7)	2.3,5.1	17 (4.5)	3.0,6.0	43 (11.7)	9.3,14.1	0.578	< 0.001^b^
**≤13 years**	26 (6.2)	4.5, 7.9	35 (9.0)	7.0,11.0	49 (13.9)	11.4,16.4	0.131	< 0.001^b^
**> 13 years**	55 (17.0)	14.3,19.7	57 (16.7)	14.0,19.4	74 (21.1)	18.1,24.1	0.914	0.182
**Current tobacco use**	25 (3.4)	2.1,4.7	36 (5.0)	3.4,6.5	29 (4.1)	2.6,5.5	0.131	0.446
**Male**	23 (6.4)	4.6,8.1	30 (9.0)	6.9,11.0	21 (6.7)	4.9,8.5	0.204	0.899
**Female**	2 (0.5)	0.0,1.0	3 (0.8)	0.2,1.4	7 (1.9)	0.9,2.9	0.652	0.102
**≤13 years**	12 (2.9)	1.7,4.1	15 (3.9)	2.5,5.3	10 (2.8)	1.6,4.0	0.410	0.976
**> 13 years**	12 (3.8)	2.4,5.2	21 (6.2)	4.5,7.9	19 (5.6)	3.9,7.3	0.155	0.275
**Tobacco cessation intention among current smokers**	12 (48.0)	44.4,51.6	12 (34.3)	30.9,37.7	8 (30.8)	27.4,34.2	0.287	0.211
**Lifetime illicit substances use**	4 (0.5)	0.0,1.0	6 (0.8)	0.2,1.4	4 (0.6)	0.03,1.2	0.523	0.425
**Male**	3 (0.8)	0.2,1.4	4 (1.2)	0.4,2.0	3 (0.9)	0.2,1.6	0.655	0.890
**Female**	,	0.0,0.4	2 (0.5)	0.0,1.0	1 (0.3)	0.0,0.7	0.994	0.994
**≤13 years**	2 (0.5)	0.0,1.0	3 (0.8)	0.2,1.4	–	–	0.600	0.994
**> 13 years**	1 (0.3)	0.0,0.7	3 ((0.9)	0.2,1.6	4 (1.2)	0.4,2.0	0.371	0.239
**Purchase of illicit substances**	14 (1.9)	0.9,2.9	51 (7.0)	5.2,8.8	32 (4.6)	3.1,6.1	< 0.001^a^	0.005^b^
**Male**	8 (2.3)	1.2,3.4	30 (8.9)	6.9,10.9	16 (5.1)	3.5,6.7	< 0.001^a^	0.055^b^
**Female**	6 (1.6)	0.7,2.5	21 (5.5)	3.9,7.1	15 (4.2)	2.7,5.7	0.006^a^	0.043^b^
**≤13 years**	10 (2.4)	1.3,3.5	31 (8.0)	6.1,9.9	15 (4.3)	2.8,5.8	0.001^a^	0.143
**> 13 years**	4 (1.3)	0.5,2.1	20 (5.8)	4.1,7.5	16 (4.6)	3.1,6.1	0.004^a^	0.018^b^
**Lifetime inhalants use**	6 (0.8)	0.2,1.4	22 (3.0)	1.8,4.2	40 (5.7)	4.0,7.4	0.004^a^	< 0.001^b^
**Male**	2 (0.6)	0.1,1.1	10 (2.9)	1.7,4.1	15 (4.7)	3.1,6.2	0.015^a^	0.001^b^
**Female**	4 (1.0)	0.3,1.7	12 (3.1)	1.9,4.3	25 (6.9)	5.0,8.7	0.044^a^	< 0.001^b^
**≤ 13 years**	1 (0.2)	0.0,0.5	12 (3.1)	1.9,4.3	26 (7.4)	5.5,9.3	0.013^a^	0.001^b^
**> 13 years**	5 (1.6)	0.7,2.5	10 (2.9)	1.7,4.1	13 (3.8)	2.4,5.2	0.249	0.090^b^
**Tobacco use among**								
**Father**	363 (49.1)	45.5,52.7	335 (45.8)	42.2,49.4	337 47.3)	43.6,51.0	0.215	0.511
**Mother**	4 (0.5)	0.0,1.0	13 (1.8)	0.8,2.7	4 (0.6)	0.03,1.2	0.036^a^	0.956
**Siblings**	108 (14.6)	12.1,17.1	99 (13.5)	11.1,15.9	108 (15.2)	12.6,17.8	0.562	0.759
**Illicit substances use among**								
**Parents**	5 (0.7)	0.1,1.3	4 (0.5)	0.0,1.0	6 (0.9)	0.2,1.6	0.757	0.690
**Siblings**	2 (0.3)	0.0,0.7	2 (0.3)	0.0,5.2	1 (0.1)	0.0,0.3	0.987	0.604
**Friends**	19 (2.6)	1.5,3.7	43 (5.9)	4.2,7.6	43 (6.1)	4.3,7.8	0.002^a^	0.001^b^
**Current psychological symptoms**								
**Depressive mood**	165 (22.0)	19.0,25.0	173 (23.0)	20.0,26.0	190 (26.6)	23.4,29.8	0.895	0.115
**Anxiety**	274 (36.5)	33.1,39.9	305 (40.6)	37.1,44.1	300 (42.0)	38.4,45.6	0.263	0.101
**Insomnia**	514 (68.4)	65.1,71.7	497 (66.2)	62.8,69.6	497 (69.5)	66.1,72.9	0.157	0.433
**Stress**	169 (22.5)	19.5,25.5	238 (31.7)	28.4,35.0	208 (29.1)	25.8,32.4	< 0.001^a^	0.019^b^

***CI*** Confidence Interval

“^a^”: significant difference between 2014 and 2015

“^b^”: significant difference between 2014 and 2016

**Fig. 1:**
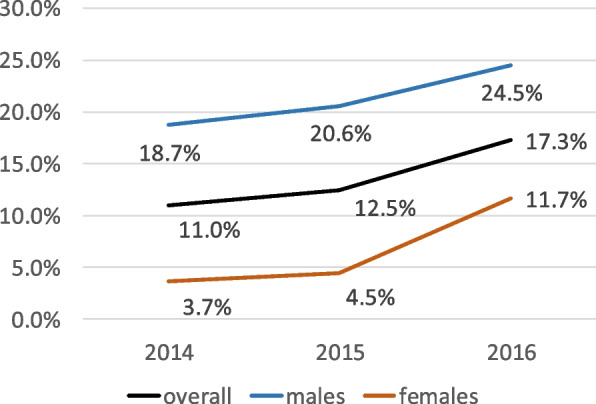
Evolution of lifetime tobacco use between 2014 and 2016 among the participants according to their sex

**Fig. 2:**
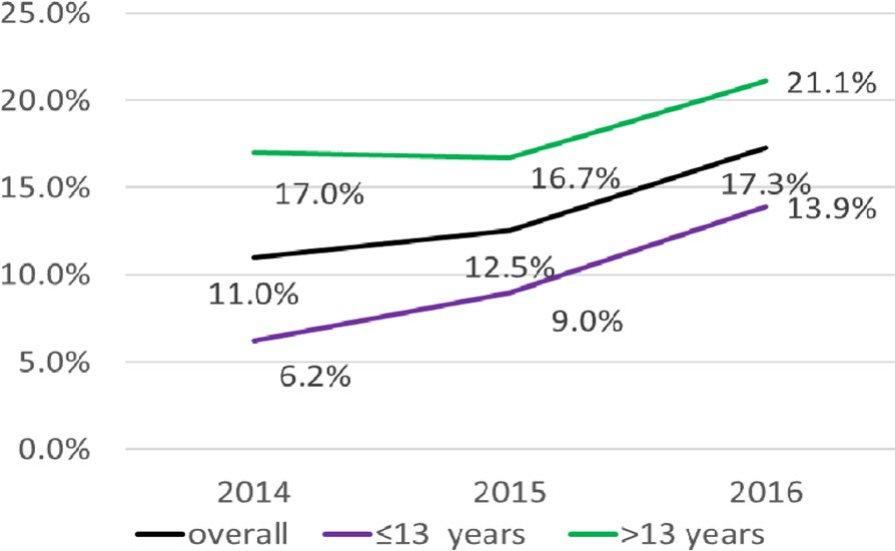
Evolution of lifetime tobacco use between 2014 and 2016 among the participants according to their age

Contrary to current cigarette smoking, which was stable among participants, lifetime tobacco use rose significantly in 2016 (17.3%) compared to 2014 (11.0%) (*p* = 0.001). Females and pupils under 14 years were the most concerned by this trend (Figs. 1 and 2). However, the prevalence level of both: current cigarette smoking and lifetime tobacco use remained higher among males and adolescents above 13 years across the three surveys (Table 1).

The age of tobacco use onset rose from 10.9 (±2.8) years in 2014 to 11.5 (± 2.5) years (*p* <  0.001) in 2015 and to 11.4 (±2.7) years (p <  0.001) in 2016. Otherwise, 38, 30.6 and 30.8% of tobacco users began tobacco consumption before the age of 11 years in 2014, 2015 and 2016 respectively.

The prevalence of lifetime illicit substances use varied between 0.5% in 2014, 0.8% in 2015 and 0.6% in 2016 without statistically significant difference across the three surveys (Table 1). However, frequency of illicit substances purchase in the middle school was higher and rose significantly from 1.9% in 2014 to 7% in 2015 (*p* <  0.001) and to 4.6% in 2016 (*p* = 0.005). The increase of illicit substances purchase was most important among those over 13 years whereas the rate of illicit substances purchase was the highest in males. Among the participants’ friends, illicit substances use rose from 2.6% in 2014 to 5.9% in 2015 (*p* = 0.002) and to 6.1% in 2016 (*p* = 0.001) (Table 1).

The mean age of illicit substances use onset went down from 14.0 (±1.7) years in 2014 to 12.2 (±2.9) years in 2015 (*p* = 0.337) and to 10.9 (±2.2) years in 2016 (*p* = 0.028). Indeed, proportion of illicit substances users who began the consumption before the age of 11 years rose from 0% in 2014 to 20% in 2015 to reach 52.4% in 2016.

Concerning inhalant products, their use increased significantly from 0.8% in 2014 to 3.0% in 2015 (*p* = 0.004) and to 5.7% in 2016 (p <  0.001). This increase was the highest among males and pupils under the age of 14 years. While inhalants were more frequently used by females across the three surveys (Table 1).

Male gender, age above 13, depressive mood, current anxiety problem, lifetime illicit substances use, lifetime inhalant products use, tobacco use among the father, tobacco use among siblings and illicit substances use among friends were significantly associated with higher prevalence of lifetime tobacco use among participants across the 3 years of survey (Table 2).

**Table 2:** Results of the analysis for lifetime tobacco use according to the individual characteristics of the participants and substances use in their entourage across 2014, 2015 and 2016

	*p*-value	OR^*^ [95% CI]
**Individual characteristics**		
**Age**	< 0.001	
**> 13 years**		2.1 [1.6,2.7]
**≤ 13 years**		1
**Sex**	< 0.001	
**Male**		3.9 [2.9,5.2]
**Female**		1
**Current psychological problems**		
**Depressive mood**	0.008	
**Yes**		1.4 [1.1,1.8]
**No**		1
**Anxiety**	< 0.001	
**Yes**		1.7 [1.3,2.3]
**No**		1
**Stress**	0.053	
**Yes**		1.3 [1,1.7]
**No**		1
**Insomnia**	0.002	
**Yes**		0.6 [0.5,0.8]
**No**		1
**Lifetime inhalants use**	0.042	
**Yes**		1.8 [1.1,3.3]
**No**		1
**Lifetime illicit substances use**	< 0.001	
**Yes**		9.1 [3.1,26.7]
**No**		1
**Substances use in the entourage**		
**Tobacco use among the father**	< 0.001	
**Yes**		1.6 [1.2,2.1]
**No**		1
**Tobacco use among the mother**	0.144	
**Yes**		2.1 [0.7,5.9]
**No**		1
**Tobacco use among siblings**	< 0.001	
**Yes**		2.0 [1.4,2.7]
**No**		1
**Illicit substances use among parents**	0.855	
**Yes**		1.1 [0.2,5.2]
**No**		1
**Illicit substances use among sibling**	0.095	
**Yes**		4.6 [0.8,28.2)
**No**		1
**Illicit substances use among friends**	< 0.001	
**Yes**		2.9 [1.8,4.5]
**No**		1

***CI*** Confidence Interval

OR* Odds ratio adjusted for the survey year

After multivariate analysis, the male sex followed by lifetime illicit substances use, age above 13, lifetime inhalant products use, tobacco use among father, tobacco use among siblings and current anxiety problem were revealed to be the most influential factors on lifetime tobacco use among participants across the 3 years of survey (Table 3).

**Table 3:** Result of the binary logistic regression analysis for the characteristics related to lifetime tobacco use among the participants across 2014, 2015 and 2016

Variables	*p*-value	OR [95% CI]	*p*-value	aOR [95% CI]
**Survey year**				
**2014**	**–**	**1**	**–**	**1**
**2015**	**0.358**	**1.2 [0.8,1.6]**	**0.955**	**1.0 [0.7,1.4]**
**2016**	**0.001**	**1.7 [1.2,2.3]**	**0.002**	**1.7 [1.2,2.4]**
**Age**	**< 0.001**		**0.000**	
**> 13 years**		2.1 [1.6,2.7]*		**2.3 [1.7,3.1]**
**≤ 13 years**		**1**		**1**
**Sex**	**< 0.001**		**< 0.001**	
**Male**		3.9 [2.9,5.2]*		**4.4 [3.2,6.1]**
**Female**		**1**		**1**
**Current anxiety problem**	**< 0.001**		**< 0.000**	
**Yes**		1.7 [1.3,2.3]*		**1.8 [1.4,2.4]**
**No**				
**Lifetime illicit substances use**	**< 0.001**		**0.033**	
**Yes**		9.1 [3.1,26.7]*		**3.9 [1.1, 13.8]**
**No**		**1**		**1**
**Lifetime inhalant use**	**0.042**		**0.015**	
**Yes**		1.8 [1.1,3.3]*		**2.2 [1.2,4.3]**
**No**		**1**		**1**
**Tobacco use among the father**	**< 0.001**		**0.015**	
**Yes**		1.6 [1.2,2.1]*		**2.2 [1.2,4.3]**
**No**				
**Tobacco use among siblings**	**< 0.001**	**1**	**0.003**	**1**
**Yes**		2.0 [1.4,2.7]*		**1.7 [1.2,2.4]**
**No**		**1**		**1**

*CI* Confidence interval

*: Odds ratio adjusted for the survey year

***aOR*** Odds ratio adjusted for all the variables in the table

## Discussion

lifetime tobacco use among middle school pupils in Sousse, Tunisia is in expansion especially among females and those younger than 14 years. Predictors of lifetime tobacco use among young adolescents of Sousse were male sex, age above 13 years, lifetime illicit substances use, lifetime inhalants use, tobacco use among the father, tobacco use among siblings and current problem of anxiety.

Compared to the 2010 and the 2017 national data highlighted among adolescents aged between 13 and 15 years, the 3 surveys revealed lower prevalence levels of both lifetime and current cigarette smoking among participants (aged between 11 and 16 years) [15]. In 2004, in the neighboring governorate of Monastir, among adolescents aged between 10 and 19 years, the overall prevalence of cigarette use during the past year was at 16.0% [23]. Further multi-centric studies on tobacco use among Tunisian adolescents would clarify whether our result is related to a regional specificity. Nonetheless, the level of tobacco use among young adolescents in Tunisia remains not far from that reported recently in Morocco and Lebanon. However, it was higher than that reported in Egypt and United states but lower than that reported in Algeria and European countries [24–26].

Both lifetime tobacco use and current cigarette smoking were more frequent among males and those older than 13 years joining recent national studies results underlined among middle schoolchildren (13-15 years) and high schoolchildren (15-18 years) [15, 27]. In developed countries, tobacco use increases also with greater age. However, there is no longer sex difference concerning this risk behavior [24, 27, 28].

The current study revealed that contrary to current cigarette smoking, lifetime tobacco use rose significantly between 2014 and 2016 which reflects the extent of tobacco marketing. This rising was more important among females which joins the results of the national study led among 13-15 years adolescents in 2017 [15] and suggests that females are targeted by tobacco industry. In developed countries, there is an opposite trend with an overall decrease in tobacco use over the last decades [24, 25]. Success stories from these countries should guide future policies of tobacco prevention in Tunisia.

Tobacco industry seems to target the youngest adolescents. In fact, almost one third of tobacco users in the three surveys began tobacco consumption before the age of 11 years which joins the 2017 national survey result [15]. This proportion of early tobacco use initiation exceeded that found recently among young European adolescents [24]. Future prevention actions of tobacco use in Tunisia should target youth as early as possible.

Having over the third of tobacco users who intent to quiet tobacco corroborates the result of the 2017 national study [15] and emphasis the necessity of providing affordable tobacco cessation service to young adolescents.

Concerning lifetime use of illicit substances, the prevalence level across the 3 years of survey was not far from that reported in previous Tunisian studies [21, 29] and in some southern Mediterranean countries but it remains lower than that in United States and northern Mediterranean countries [24, 25, 29].

Males outperformed females concerning purchase and lifetime use of illicit substances which joins previous national studies [29] and other recent studies led in other North African, Middle East and European countries [24, 26] while in United states for example this sex gap became smaller [25].

Lifetime illicit substances use did not rose across the 3 years of survey among participants. Nonetheless, purchase of illicit substances in the middle schools rose significantly. This discordance is due probably to underreporting of illicit substances use by participants. At the national level, illicit substances use rose between 2013 and 2017 among high school pupils which is in favor of illicit substances use expansion in Tunisia [29]. Globally, trends of illicit substances use vary according to geographical regions. For example, in United States, illicit substances use increased among adolescents over the last decades. Whereas a general decrease was seen over the last decades in European countries [24, 25].

The age of illicit substances initiation stepped back across the 3 years of survey. However, in the 2017 national study led among high school pupils, lifetime use of illicit substances after the age of 14 years was more frequent [29]. Further studies would elucidate whether illicit substances would become the first substances used instead of tobacco in Tunisia like what happened in United States for example [11].

Prevalence of inhalants use among participants across the 3 surveys was not far from that of 3.8% revealed by the 2017 national study among high schools students [29]. Inhalants use was more frequent among female participants and those under 14 years. While it was more frequent among males in the 2017 national study [29]. Globally, frequency and distribution of inhalants use varies between countries [26]. Nonetheless it is usually used during early adolescence [30, 31].

Inhalants use increased significantly during the 3 years of survey among participants especially among males and those less than 14 years. In Europe, recent studies showed stability of inhalants use among females and a decrease among males [24] while in United states, inhalants use declined over the last decades [30].

Focusing on mental health, across the three surveys, almost one in four participants reported current mood disturbance and one in three participants reported current feeling of stress. Worldwide, frequency of mental health disorders among adolescents is between 10 and 20% [32]. National study with validated measure tool is required to determine the importance of such health problems in Tunisian adolescents.

Depressive mood increased significantly among females while anxiety rose significantly among pupils above the age of 14. Actually, gender disparity concerning mental health problems is frequently reported worldwide [33] and the half of mental illnesses begin usually by the age of 14 [32].

Male sex was the most influential factor on lifetime tobacco use among the adolescents of the region of Sousse across 2014, 2015 and 2016. In fact, sex inequality in tobacco use depends on cultures and tobacco products [27, 34]. In Eastern Mediterranean countries such Tunisia, tobacco use is more common among males [27, 34]. However, this gender sex is narrowing in these countries [27]. Females seem to represent a good marketing opportunity for tobacco industry.

The second influential factor on lifetime tobacco use among participants was illicit substances use. This result joins those of a larger study led in the region of Sousse in 2014 and the last national study led among high school pupils [21, 29]. The sequence: early cigarette smoking followed by later illicit drugs use is frequently reported in the literature [35, 36]. Nevertheless, the cross-sectional nature of the current study could not allow making conclusions about whether tobacco use is a cause or a consequence of illicit substances use among participants.

Another predictor of lifetime tobacco use among participants was: inhalant products use. Previous longitudinal studies showed that inhalants represent an intermediate drug class between legal drugs and illegal drugs [37]. A longitudinal study would identify the gateway substance among Tunisian adolescents.

Having more than 13 years was an independent risk factor of lifetime tobacco use among participants. Previous epidemiological studies corroborate this finding and showed that prevalence rate of tobacco use increases along with adolescence to reach a peak among young adults and declines thereafter [38, 39].

Current anxiety was another predictor of lifetime tobacco use among participants. In fact, anxiety disorders may increase risk for nicotine dependence and nicotine dependence increases risks for the development of anxiety disorders [40]. In general, co-occurrence of substance use and mood disorders is frequently reported [41]. Policy makers should pay attention to mental health problems when planning for substances use prevention actions.

Tobacco use among the father and tobacco use among siblings were independently associated with lifetime tobacco use. Previous studies reported that a greater number of smokers in home represent significant risk factor for smoking initiation [42–44]. Providing smoke-free environment to young people is therefore recommended to prevent early tobacco use onset.

Results of the current study suggest that the current national tobacco prevention program should be strengthened and expanded to cover other substances use issues. Prohibiting the sale of tobacco products around schools would reduce their affordability to pupils. Besides, the reinforcement of the existing legislative measures related to secondhand smoking, the sale of tobacco products to children and illicit substances trafficking are necessary. In the schools, the implementation of a comprehensive national prevention program of substances use should no longer be delayed. This program should focus on the wellbeing and the development of soft skills among schoolchildren. Involving parents, teachers, directors and the administrative personal of the schools in addition to peers’ education would increase the efficacy of this program. For secondary prevention of tobacco use, the affordability of tobacco cessation services should be increased for adolescents by building capacity on tobacco prevention and control among school health professionals.

The current results should be interpreted with taking into consideration the limits of the study. Firstly, because of the cross-sectional nature of the three surveys, it was not possible to report causal relationships but only simple associations. Besides, this study was led in middle schools, consequently, inference on “out of school” adolescents could not be possible. Finally, substances use were self-reported which could result in social desirability bias. However, data were collected anonymously and participation was voluntary. Furthermore, repetitive measure with the same questionnaire would reduce this bias.

## Conclusion

Lifetime tobacco use among middle school pupils in Sousse, Tunisia is in expansion especially among females and the youngest ones. To stop this trend, it is necessary to implement national comprehensive substances use prevention program since early adolescence. More attention should be paid to secondhand smoking and mental health problems among the Tunisian young adolescents.

## Data Availability

The datasets supporting the conclusions of this article are not publicly available due to limitations of ethical approval involving the participant’s data and anonymity but are available from the corresponding author on reasonable request.

## Abbreviations

CO: Carbon Monoxide
OR: Odds ratio
CI: Confidence Interval
